# Supplementary material for: Genomic Analysis of Clostridium perfringens BEC/CPILE-Positive, Toxinotype D and E Strains Isolated from Healthy Children
Source: Toxins (Basel). 2019 Sep 19;11(9):543. doi: 10.3390/toxins11090543 (PMC6783817; doi:10.3390/toxins11090543)
Supplement: Supplementary file 1 [file toxins-11-00543-s001.pdf]

Supplementary Materials: Genomic Analysis of *Clostridium perfringens* BEC/CPILE-positive, Toxinotype D and E Strains Isolated from Healthy Children

Raymond Kiu, Kathleen Sim, Alex Shaw, Emma Cornwell, Derek Pickard, J. Simon Kroll and Lindsay J. Hall

Table S1. Clinical data on individual preterm infants from which *C. perfringens* strains were isolated.

	Individual	IQ1	IQ2	IQ3
Post-birth (NICU)	Birth mode	C-section	C-section	C-section
	Disease history	No NEC or sepsis	Initially diagnosed with very early NEC two days post birth—but later confirmed as non-NEC.	Diagnosed with NEC (Bell stage 2), was transferred to another unit but was treated conservatively.
	Antibiotic use	No antibiotic use	No data	IV antibiotics
Post-NICU (home)	Health conditions	Does not have a questionnaire completed	Healthy child, no courses of antibiotics required since leaving NICU. No regular medications (except for multivitamins), no allergies, pets or smokers at home. Takes probiotics (in yoghurts).	One course of antibiotics for a chest infection since leaving NICU, but no antibiotics in the month preceding sample collection. No long-term health conditions other than eczema. No pets or smokers at home. No probiotic use.
Hospital location		London, UK	London, UK	London, UK

Table S2. Variable sites comparison in 864 amino acid multi-sequence alignments in between *C. perfringens* type E strains.

Strain	cp508.17	cp515.17	JGS1987	IQ2
cp508.17	0	1	143	1
cp515.17	1	0	142	0
JGS1987	143	142	0	142
IQ2	1	0	142	0

Table S3. Blastn query coverage and identity data on BEC-positive *C. perfringens* IQ3 genome assembly using reference *becAB* genes via ABRicate.

START	END	GENE	COVERAGE	GAPS	%COVERAGE	%IDENTITY
12,828	14,087	*becA*	1-1260/1260	0/0	100.00	100.00
14,106	16,505	*becB*	1-2400/2400	0/0	100.00	100.00

Table S4. Identity of alveolysin gene *alv* in *C. perfringens* isolate IQ3.

START	END	GENE	COVERAGE	GAPS	%COVERAGE	%IDENTITY	ACCESSION
50,535	52,076	*alv*	1-1542/1542	0/0	100.00	99.74	NZ_SRYQ01000017.1: c51724-50183

Table S5. RefSeq genomes used to generate *Clostridium* genus-specific database for genome annotation (Prokka). * Recently suggested to be moved to genus *Clostridioides*. # Recently suggested to be classified as genus *Paeniclostridium*.

	RefSeq Genome	Strain	NCBI Accession
1	*C. perfringens*	1207_CPER	SAMN03197169
2	*C. perfringens*	ATCC13124	SAMN02604008
3	*C. perfringens*	B_ATCC3626	SAMN02436295
4	*C. perfringens*	CBA7123	SAMD00057672
5	*C. perfringens*	C_JGS1495	SAMN02436294
6	*C. perfringens*	CP15	SAMN06252080
7	*C. perfringens*	CPE_F4969	SAMN02436168
8	*C. perfringens*	D_JGS1721	SAMN02436277
9	*C. perfringens*	E_JGS1987	SAMN02436167
10	*C. perfringens*	FORC003	SAMN03140316
11	*C. perfringens*	FORC025	SAMN04209542
12	*C. perfringens*	JFP718	SAMN05323879
13	*C. perfringens*	JFP836	SAMN05323894
14	*C. perfringens*	JJC	SAMN02317206
15	*C. perfringens*	JP55	SAMN03372134
16	*C. perfringens*	JP838	SAMN03377063
17	*C. perfringens*	LLY_N11	SAMN07624709
18	*C. perfringens*	NCTC8239	SAMN02436239
19	*C. perfringens*	13	SAMD00061119
20	*C. perfringens*	2789STDY5608889	SAMEA3545297
21	*C. acetobutylicum*	ATCC824	SAMN02603243
22	*C. baratti*	Sullivan	SAMN03222823
23	*C. botulinum*	ATCC3502	SAMEA1705919
24	*C. butyricum*	KNU-L09	SAMN04293668
25	*C. chauvoei*	JF4335	SAMEA102059668
26	*C. difficile**	630	SAMEA1705932
27	*C. difficile* *	BR81	SAMN06286529
28	*C. difficile**	E15	SAMEA3138880
29	*C. difficile**	M120	SAMEA3138368
30	*C. novyi*	NT	SAMN02603464
31	*C. paraputrificum*	LH025	SAMEA104287982
32	*C. pasteurianum*	DSM525	SAMN02990135
33	*C. sordellii#*	AM370	SAMN04263499
34	*C. tertium*	LH009	SAMEA104287969
35	*C. tetani*	E88	SAMN02603289

Table S6. Accession numbers of plasmid sequences used in this study.

Organism	Plasmid Name	RefSeq accesion	GenBank Accession
Clostridium perfringens	pBCNF5603	NC_006872.1	AB189671
Clostridium perfringens	pCBA7123-01	NZ_AP017631.1	AP017631
Clostridium perfringens	pCP-OS1	NC_023917.1	AP013033
Clostridium perfringens	pCP-TS1	NC_023918.1	AP013034
Clostridium perfringens	pCP13	NC_003042.1	AP003515
Clostridium perfringens	pCP15_1	NZ_CP019568.1	CP019568
Clostridium perfringens	pCP15_2	NZ_CP019569.1	CP019569
Clostridium perfringens	pCP15_3	NZ_CP019570.1	CP019570
Clostridium perfringens	pCP15_4	NZ_CP019571.1	CP019571
Clostridium perfringens	pCP8533S12	NC_019358.1	AB736082
Clostridium perfringens	pCP8533etx	NC_011412.1	AB444205
Clostridium perfringens	pCPF4969	NC_007772.1	AB236336
Clostridium perfringens	pCPF5603	NC_007773.1	AB236337
Clostridium perfringens	pCPPB-1	NC_015712.1	AB604032
Clostridium perfringens	pCW3	NC_010937.1	DQ366035
Clostridium perfringens	pCpb2-CP1	NC_019687.1	JQ655732
Clostridium perfringens	pDel1_1	NZ_CP019577.1	CP019577
Clostridium perfringens	pDel1_2	NZ_CP019578.1	CP019578
Clostridium perfringens	pDel1_4	NZ_CP019580.1	CP019580
Clostridium perfringens	pDel1_3	NZ_CP019579.1	CP019579
Clostridium perfringens	pF262A	NZ_CM001478.1	CM001478
Clostridium perfringens	pF262B	NZ_CM001479.1	CM001479
Clostridium perfringens	pF262C	NZ_CM001480.1	CM001480
Clostridium perfringens	pF262D	NZ_CM001481.1	CM001481
Clostridium perfringens	pFORC3	NZ_CP009558.1	CP009558
Clostridium perfringens	pIP404	NC_001388.1	M32882
Clostridium perfringens	pJFP55F	NZ_CP013041.1	CP013041
Clostridium perfringens	pJFP55G	NZ_CP013042.1	CP013042
Clostridium perfringens	pJFP55H	NZ_CP013043.1	CP013043
Clostridium perfringens	pJFP55J	NZ_CP013044.1	CP013044
Clostridium perfringens	pJFP55K	NZ_CP013045.1	CP013045
Clostridium perfringens	pJFP838A	NZ_CP013615.1	CP013615
Clostridium perfringens	pJFP838C	NZ_CP013040.1	CP013040
Clostridium perfringens	pJFP838D	NZ_CP013039.1	CP013039
Clostridium perfringens	pJFP838E	NZ_CP013038.1	CP013038
Clostridium perfringens	pJIR3536	NC_025042.1	JN689219
Clostridium perfringens	pJIR3537	NC_019259.1	JN689220
Clostridium perfringens	pJIR3843	NC_019258.1	JN689218
Clostridium perfringens	pJIR3844	NC_019257.1	JN689217
Clostridium perfringens	pLLY_N11_2	NZ_CP023412.1	CP023412
Clostridium perfringens	pLLY_N11_3	NZ_CP023413.1	CP023413
Clostridium perfringens	pNetB-NE10	NC_019688.1	JQ655731
Clostridium perfringens	pSM101A	-	CP000313
Clostridium perfringens	pSM101B	-	CP000314

Table S7. Accession numbers of toxin genes used in this study.

	Gene Name	Product	Protein Function	NCBI Accession
1	*plc*	Phospholipase	Toxin	D63911.1
2	*cpe*	Enterotoxin CPE	Toxin	M98037.1
3	*colA*	Microbial collagenase	Toxin	D13791.1
4	*cpb*	Beta-toxin	Toxin	KP064410.1
5	*cpb2 variant 1*	Beta-2 toxin variant 1	Toxin	AP003515.1
5	*cpb2 variant 2*	Beta-2 toxin variant 2	Toxin	CP009558.1
6	*pfo*	Perfringolysin O	Toxin	BA000016.3
7	*etx*	Epsilon-toxin	Toxin	M95206.1
8	*iap*	Iota-toxin component Ia	Toxin	NC_015712.1
9	*ibp*	Iota-toxin component Ib	Toxin	NC_015712.1
10	*netB*	Pore-forming toxin NetB	Toxin	FJ189503.1
11	*becA*	Binary enterotoxin component a	Toxin	NC_023918.1
11	*becB*	Binary enterotoxin component b	Toxin	NC_023918.1
12	*tpeL*	Peptidase	Toxin	EU848493.1
13	*netF*	NE Toxin NetF	Toxin	KJ606986
14	*lam*	Lambda toxin	Toxin	AJ439340
15	*alv*	Alveolysin	Putative toxin	TGY44713.1

Table 8. *C. perfringens* genomes retrieved from NCBI RefSeq database for phylogenetic analysis.

Assembly Accession	Strain	Assembly Accession	Strain
GCF_000009685.1	13	GCF_003181535.1	67
GCF_000013285.1	ATCC13124	GCF_003181555.1	48
GCF_000171135.1	JGS1495	GCF_003181575.1	37
GCF_000171155.1	ATCC3626	GCF_003181595.1	SAF-1
GCF_000171175.1	JGS1987	GCF_003181615.1	Pennington
GCF_000171195.1	F4969	GCF_003181655.1	NAG-NE31
GCF_000171215.1	str.NCTC8239	GCF_003181675.1	NAG-NE1
GCF_000172455.1	JGS1721	GCF_003181695.1	ITX1105-12MP
GCF_000243175.1	WAL-14572	GCF_003181705.1	GNP-1
GCF_000255475.1	F262	GCF_003181735.1	FC2
GCF_000512415.1	JJC	GCF_003181745.1	EUR-NE15
GCF_001052445.1	1207_CPER	GCF_003181795.1	98.78718-2
GCF_001304735.1	FORC_003	GCF_003181815.1	96-7415
GCF_001406415.1	2789STDY5608889	GCF_003181835.1	Warren
GCF_001414595.1	CP4	GCF_003181875.1	SOM-NE34
GCF_001546355.1	MJR7757A	GCF_003181895.1	11
GCF_001579765.1	JP55	GCF_003181955.1	NobL1
GCF_001579785.1	JP838	GCF_003181995.1	EHE-NE7
GCF_001854085.1	FORC_025	GCF_003203455.1	EHE-NE18
GCF_001949205.1	JFP718	GCF_003245405.1	SYD-NE41
GCF_001949215.1	JFP774	GCF_003350945.1	JXJA17
GCF_001949235.1	JFP728	GCF_003475625.1	AF30-3

Assembly Accession	Strain	Assembly Accession	Strain
GCF_001949245.1	JFP727	GCF_003628405.1	2016TE7641_69
GCF_001949295.1	JFP795	GCF_003849925.1	SC4-C24
GCF_001949305.1	JFP801	GCF_003849945.1	SC4-C17
GCF_001949315.1	JFP804	GCF_003850125.1	SC4-C13
GCF_001949325.1	JFP796	GCF_003990265.1	tumat
GCF_001949375.1	JFP771	GCF_004115725.1	83921_3_4
GCF_001949385.1	JFP810	GCF_004116045.1	79385-2
GCF_001949395.1	JFP826	GCF_004116125.1	79385-3
GCF_001949405.1	JFP828	GCF_004116135.1	79385-4
GCF_001949455.1	JFP829	GCF_004116145.1	79385-5
GCF_001949465.1	JFP833	GCF_004116155.1	79385-7
GCF_001949485.1	JFP834	GCF_004116165.1	79385-6
GCF_001949515.1	JFP836	GCF_004116225.1	83921_1_2
GCF_001949535.1	JFP914	GCF_004116235.1	83921_3_3
GCF_001949545.1	JFP916	GCF_004116255.1	79385-1
GCF_001949565.1	JFP921	GCF_004329595.1	13/0847-D
GCF_001949595.1	JFP922	GCF_004329605.1	13/0815-D
GCF_001949615.1	JFP923	GCF_004329615.1	13/0816-B
GCF_001949625.1	JFP941	GCF_004329675.1	13/0815-C
GCF_001949635.1	JFP961	GCF_004793785.1	NM49_B9-7
GCF_001949695.1	JFP978	GCF_005844105.1	BSD2780061688st3_G3
GCF_001949715.1	JFP980	GCF_005844665.1	1001175st1_F9
GCF_001949725.1	JFP981	GCF_006385425.1	TypeD
GCF_001949735.1	JFP982	GCF_006409095.1	JS2202
GCF_001949775.1	JFP983	GCF_006454475.1	JS5388
GCF_001949795.1	JFP992	GCF_900447035.1	NCTC9851
GCF_001949805.1	JFP986	GCF_900447075.1	NCTC3182
GCF_002012325.1	Del1	GCF_900447085.1	NCTC10578
GCF_002018235.1	CP15	GCF_900447095.1	NCTC10614
GCF_002160285.1	An185	GCF_900447105.1	NCTC8798
GCF_002161405.1	An68	GCF_900447125.1	NCTC10719
GCF_002312985.1	LLY_N11	GCF_900447135.1	NCTC11144
GCF_002355795.1	CBA7123	GCF_900461135.1	NCTC8679
GCF_003075475.1	BzA	GCF_900461145.1	NCTC8359
GCF_003181355.1	UDE95-1372	GCF_900461155.1	NCTC8449
GCF_003181395.1	WER-NE36	GCF_900461235.1	NCTC10613
GCF_003181405.1	TAM-NE46	GCF_900461295.1	NCTC8081
GCF_003181415.1	TAM-NE42	GCF_900461395.1	NCTC10240
GCF_003181455.1	TAM-NE43	GCF_900475545.1	NCTC13170
GCF_003181475.1	TAM-NE40	GCF_900478015.1	NCTC2837
GCF_003181495.1	SOM-NE35	GCF_900604525.1	cp508.17
GCF_003181505.1	TAM-NE38	GCF_900604545.1	cp515.17
